# [Corrigendum] Dual roles of protein tyrosine phosphatase kappa in coordinating angiogenesis induced by pro-angiogenic factors

**DOI:** 10.3892/ijo.2025.5772

**Published:** 2025-07-09

**Authors:** Ping-Hui Sun, Gang Chen, Malcolm Mason, Wen G. Jiang, Lin Ye

Int J Oncol 50: 1127-1135, 2017; DOI: 10.3892/ijo.2017.3884

Subsequently to the publication of the above paper, an interested reader drew to the authors' attention that the data for the PTPRK blots shown in Fig. 1B on p. 1129 were strikingly similar to data that had already appeared in a previous publication by the same authors in the journal *PLoS One*. The authors have re-examined their original data, and realize how this error occurred. The revised (and corrected) version of Fig. 1, now showing the correct data for the PTPRK blots in Fig. 1B, is shown below. The authors sincerely apologize for the error made in assembling this figure, although they confirm that this did not grossly affect either the results or the conclusions reported in this study. They also thank the Editor of *International Journal of Oncology* for granting them the opportunity to publish a Corrigendum, and apologize to the readership for any inconvenience caused.

## Figures and Tables

**Figure 1:**
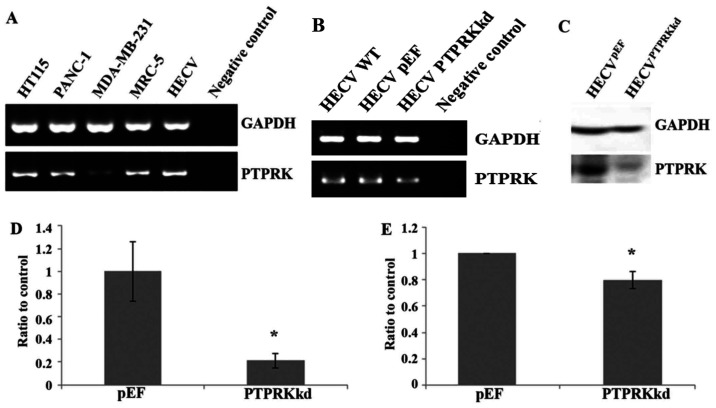
PTPRK gene expression and knockdown of PTPRK in HECV cells. (A) Expression of PTPRK mRNA in different cell lines (HT115, colon cancer cell; PANC-1, pancreatic cancer cell; MDA-MB-231, breast cancer cell; MRC-5, lung fibroblast cell; HECV, endothelial cell). Knockdown of PTPRK was seen in HECV PTPRKkd cells compared with empty plasmid control (HECV pEF cells) using RT-PCR (B), western blot analysis (C) and real-time quantitative PCR (D). (E) PTPRK protein band volume of three repeats which is normalised against corresponding internal control. The intensity shown is integrated band intensity (intensity × area) and was normalised against the corresponding GAPDH signal. ^*^P<0.05.

